# *Pseudomonas aeruginosa* adaptation in cystic fibrosis patients increases C5a levels and promotes neutrophil recruitment

**DOI:** 10.1080/21505594.2022.2028484

**Published:** 2022-01-30

**Authors:** Margalida Mateu-Borrás, Alex González-Alsina, Antonio Doménech-Sánchez, Javier Querol-García, Francisco J. Fernández, Mª Cristina Vega, Sebastián Albertí

**Affiliations:** a Instituto Universitario de Investigación En Ciencias de La Salud, Universidad de Las Islas Baleares and Instituto de Investigación Sanitaria de Les Illes Balears, Palma de Mallorca, Spain; b Centro de Investigaciones Biológicas Margarita Salas, Consejo Superior de Investigaciones Científicas, Madrid, Spain

**Keywords:** C5a, alkaline protease A, elastase B, lasR, cystic fibrosis

## Abstract

Cystic fibrosis (CF) disease is characterized by an intense airway inflammatory response mediated by neutrophils and chronic respiratory infections caused by *P. aeruginosa*. High levels of the complement component C5a, the strongest neutrophil chemoattractant molecule, are commonly found in the CF lung and have been associated with a worsening of the disease. In this study, we investigated how the isolates from CF patients modulate the levels of C5a and identified the bacterial factors involved. We demonstrated that most isolates from airway chronic infections induce the production and accumulation of C5a, an effect attributable to the loss of C5a cleavage by the exoproteases alkaline protease (AprA) and elastase B (LasB). Furthermore, we found that lack of the bacterial protease-dependent C5a degradation is due to mutations in the master regulator LasR. Thus, complementation of a non-C5a-cleaving CF isolate with a functional wild-type LasR restored its ability to express both proteases, cleave C5a and reduce neutrophil recruitment in vitro. These findings suggest that the non-cleaving C5a phenotype acquired by the LasR variants frequently isolated in CF patients may account for the strong neutrophilia and general neutrophil dysfunction predisposing toward increased inflammation and reduced bacterial clearance described in CF patients.

## Introduction

*Pseudomonas aeruginosa* is the major cause of morbidity and mortality in cystic fibrosis (CF) patients [1]. The presence of the microorganism in the CF lung is determinant for the development and progression of the disease. Initially, the microorganism colonizes the lung intermittently and, after a time, eradication is almost impossible and it becomes perpetually established. A common characteristic of the CF chronic lung infections by *P. aeruginosa* is that the establishment of the infection correlates with the emergence of a wide array of phenotypes [2,3]. *P. aeruginosa* isolated from CF patients includes clonally related variants that can be lipopolysaccharide deficient, nonflagellated, mucoid, auxotrophic or resistant to antimicrobial agents [2,3]. It is likely that this wide spectrum of phenotypes contributes to the lung destruction responsible for morbidity and mortality and is the result of the continuous adaptation of the microorganism to evade the host defense and persist in the CF lung [4].

The complement system is an essential mechanism of the host defense and a main contributor to the inflammatory response. One of the most important effectors of this system is C5a, a polypeptide of 74 amino acid residues resulting from the cleavage of C5 by C5 convertases during complement activation. C5a is the most potent chemoattractant molecule involved in the recruitment of neutrophils to the site of infection. The binding of C5a to the specific receptors C5aR1 and C5aL2 on the neutrophils surface induces a range of responses crucial for host defense, including chemotaxis, phagocytosis and respiratory burst [5]. While some C5a-induced signaling may be required for bacterial clearance, in vitro studies, experimental animal models of sepsis and clinical evidence demonstrate that an excessive amount of C5a impairs the generation of reactive oxygen species and phagocytosis of bacteria by neutrophils [6–8].

It has been shown that the CF lung fluid contains increased amounts of C5a compared with healthy controls [9,10]. More recently, Hair et al. reported that increased C5a in the sputum of CF patients is associated with lung hyperinflammation and is a marker of poor prognosis. Furthermore, *P. aeruginosa* load in the sputum correlated positively with C5a concentrations [11].

In the present study, we aimed to investigate how the chronic presence of *P. aeruginosa* in the lung of CF patients impacts on the level of C5a present in the inflamed lung and identify the bacterial factors involved in this effect.

## Materials and methods

### Bacterial strains

Three sets of *P. aeruginosa* clinical strains collected from respiratory samples were used in this study. The first set of 20 strains belongs to a larger collection previously described [12]. Their characteristics are listed in the supplemental Table S1. This set of strains included the first and last available clonally related isolate, collected with a separation interval of at least 3 years, from ten CF patients. Six additional clones from patient FQSE11 were also included in the study.

The second group of 22 isolates was collected from patients colonized by *P. aeruginosa* for at least 3 years. They represented unique clones as verified by pulsed-field gel electrophoresis [13]. The last set of 22 strains was obtained from epidemiologically unrelated patients with acute respiratory infections attended in the intensive care unit [13].

*P. aeruginosa* reference strain PA14 and the isogenic *aprA-*deficient mutant (PA14Δ*aprA*), *lasB-*deficient mutant (PA14Δ*lasB*) and *aprA* and *lasB-*deficient double mutant (PA14Δ*aprA*Δ*lasB)* were previously described [14]. The phenotype of each strain was confirmed by RT-PCR analysis of the specific *aprA* and *lasB* transcripts and mass spectrometry analysis of the proteins present in the cell-free supernatant of the bacterial growth cultures.

Bacterial cells were grown in Luria Bertani (LB) broth with shaking or in LB solidified with 1.5% agar that was supplemented with 0.1% arabinose (w/v) and carbenicillin (100 μg/ml) when they were transformed with plasmid pMMB1. In some experiments, bacterial cells were grown in artificial sputum medium [15].

### Human reagents

A pool of human sera was obtained from blood of healthy volunteers. A pool of human bronchoalveolar lavage fluid (BALF) was collected using standard procedures [16]. BALF was centrifuged to discard cells, concentrated 50-fold and stored at −70°C until its use. Recombinant human C5a (including the C-terminal Arg74) expressed in bacteria as a glutathione-*S*-transferase (GST) fusion and purified to homogeneity by glutathione-affinity chromatography, GST removal by digestion with the rhinovirus 3 C protease, and size-exclusion chromatography. C5a was concentrated to 1 mg/ml, snap frozen in small (30 μl) aliquots and stored away at −80°C until used.

### Ethics statement

Human samples were taken after informed consent of each participant. The study was aligned with the Helsinki Declaration and was approved by the Institutional Review Board of Hospital Universitario Son Espases (Palma de Mallorca, Spain) and the Regional Ethics Committee of Illes Balears.

### Analysis of C5a

Analysis of C5a was carried out by Western blotting or enzyme-linked immunosorbent assays (ELISA). In Western blotting experiments, 3 × 10^9^ colony forming units (CFU) were incubated at different times at 37°C in 1 ml of human serum at 10% in Phosphate-Buffered Saline (PBS). Samples were boiled in loading buffer (50 mM Tris-HCl pH 6.8, 10% glycerol, 2% SDS, 0.01% bromophenol blue) and subjected to Western blot analysis with a mouse monoclonal antibody that recognizes C5a (Abcam). Blots were analyzed by densitometry with the GS-800 Calibrated densitometer (BioRad).

C5a was also quantified using the Human C5a/Complement C5a ELISA Kit (Sigma-Aldrich) following the manufacturer’s instructions.

### C5a cleavage assays

For the C5a cleavage assays we used the bacterial cells or the cell-free supernatant of *P. aeruginosa* stationary phase cultures grown at 37°C in LB broth. The culture supernatant was separated from the bacterial cells by centrifugation and then via filtration through a 0.22-μm-pore-size membrane (Millipore). Recombinant human C5a (1 ng/μl) was incubated with bacterial cells or the cell-free supernatant and, subsequentially, the presence of C5a was analyzed by Western blot as described above.

### RNA extraction and real time PCR

Total cellular RNA was isolated from stationary phase cultures of *P. aeruginosa* using the Qiagen RNeasy Mini Kit (Qiagen) according to the manufacturer’s instructions. After DNase treatment with Turbo DNA-*free* Kit (ThermoFisher), one step reverse transcription and real-time PCR amplification was performed on 50 ng of purified RNA by using the QuantiTect SYBR green RT-PCR kit (Qiagen) with an Eco Real-Time PCR System (Illumina). Negative controls consisting in RNase-free water were added to detect possible DNA contamination. Data were analyzed with EcoStudy Software V.5.0. Gene expression was normalized using the housekeeping gene *rpsL*. The results were always referenced against PA14 expression.

Primers used for the amplification of those genes are described in supplemental Table S2. The level of gene expression was calculated using comparative 2^-Ct method [17].

### DNA procedures

For the sequencing of *lasR*, the gene was obtained by PCR amplification of the genomic DNA from the clinical isolates listed in Table S1 and sequenced with primers LasR Seq-F and LasR Seq-R. For the cloning of *lasR*, the gene was amplified by PCR using the genome of the clinical isolate FQSE11-0603 as template and the primers LasR-F/LasR-R. The amplicon containing *lasR* was cut with EcoRI/HindIII and cloned into the pJPO4 vector [18] to give plasmid pMMB1, which was transformed into *P. aeruginosa* FQSE-11-1010 by electroporation.

Primer sequences are shown in supplemental Table S2. All molecular biology procedures were carried out following standard protocols [19].

### Polymorphonuclear migration assays

Migration assays were conducted using fresh human polymorphonuclear cells (PMNs) isolated by dextran sedimentation and Ficoll-Histopaque density gradient centrifugation [20]. Chemotactic migration was assessed in 24-well Transwell® plates with 5 μm pore size polycarbonate filters (Corning). Approximately 2 × 10^5^ PMNs resuspended in RPMI-1640 were loaded in the upper chambers, while lower chambers contained RPMI-1640, recombinant human untreated C5a (100 nM), or C5a treated for 4 h at 37°C with the cell-free supernatant of bacterial stationary phase cultures from different strains. After 2 h at 37°C, the number of PMNs recovered from lower chambers was determined using a cell counter. The percent PMNs transmigration was defined as the percentage of PMNs in the lower chamber compared to the total number of PMNs loaded in the upper chamber.

## Results

### *P.aeruginosa* cleaves C5a, a capacity lost by the isolates from chronic airway infections

**Figure 1.**
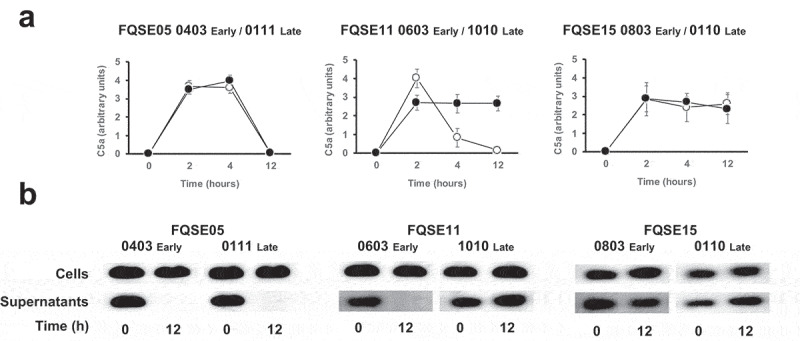
**Analysis of the production and cleavage of C5a by *P. aeruginosa* strains isolated from CF patients with chronic airway infections**. (a). Bacterial cells from three pairs of clonally related isolates, including the earliest (early, white circles) and the latest (late, black circles) isolate, recovered from three chronically infected CF patients were incubated in human serum (10%) and the amount of C5a produced was determined at different times by Western blot using a monoclonal antibody that recognizes C5a. Quantification of the C5a band was carried out by densitometric analysis. Results are the mean values and the standard deviation obtained from three independent experiments for each strain. (b) Purified recombinant human C5a (20 ng) was incubated for 1 h at 37°C with the cells or the cell-free supernatant from stationary cultures of *P. aeruginosa* strains isolated from CF patients with chronic airway infections. Proteins were separated and subjected to a Western blot with a monoclonal antibody that recognizes C5a.

To begin understanding the impact of the chronic presence of *P. aeruginosa* on the levels of C5a in the lung, we first determined the production of C5a through the activation of complement’s terminal pathway by three pairs of clonally related isolates, including the earliest (early) and the latest (late) available isolates recovered from three chronically infected CF patients. Bacterial isolates were incubated in human serum at 10% in PBS, to simulate the level of the complement components present in the bronchoalveolar lavage fluid of the human lung [16], and the amount of C5a was determined at different time points by densitometric analysis of the specific band detected by Western blotting with a monoclonal antibody that recognizes C5a (Figure 1a). The amount of C5a present in the sera incubated with bacteria increased rapidly, reaching the maximum level after 2 h. C5a was not detected in sera incubated with LB alone as control (data not shown). Interestingly, the amount of C5a present in the sera incubated with the early and late isolates from the patient FQSE05 (FQSE05-0403 and 0111) and the early isolate from the patient FQSE11 (FQSE11-0603) decreased over the time and was almost undetectable at 12 h, while in the sera incubated with the isolates from patient FQSE15 (FQS15-0803 and 0110) and the late isolate from the patient FQSE11 (FQSE11-1010) the amount of C5a remained stable. The reference strain PA14 exhibited a pattern similar to that observed with the isolates from patient FQSE05 (Fig. S1).

There was no significant difference in the number of viable bacteria at different times between isolates, confirming that differences in C5a levels were not due to the bacterial load (data not shown).

This finding led us to hypothesize that CF early isolates FQSE05-0403 and FQSE11-0603 and the late isolate FQSE05-0111 cleaved C5a, a capacity not exhibited by the other CF isolates used in this experiment.

To investigate this hypothesis, we tested whether the bacterial cells or the cell-free supernatants of stationary bacterial cultures of these isolates were able to degrade recombinant human C5a (Figure 1b). Bacterial cells were unable to cleave C5a independently of the strain tested. Conversely, no C5a was detected in the sample treated with the cell-free supernatant from cultures of FQSE05-0403, FQSE05-0111 and FQSE11-0603, whereas the C5a treated with the supernatant from cultures of the rest of the chronic isolates remained intact as the control of untreated C5a. This observation suggests that C5a was cleaved by proteases secreted by *P. aeruginosa*, which are absent or not functional in some strains isolated from chronic respiratory infections.

**Figure 2.**
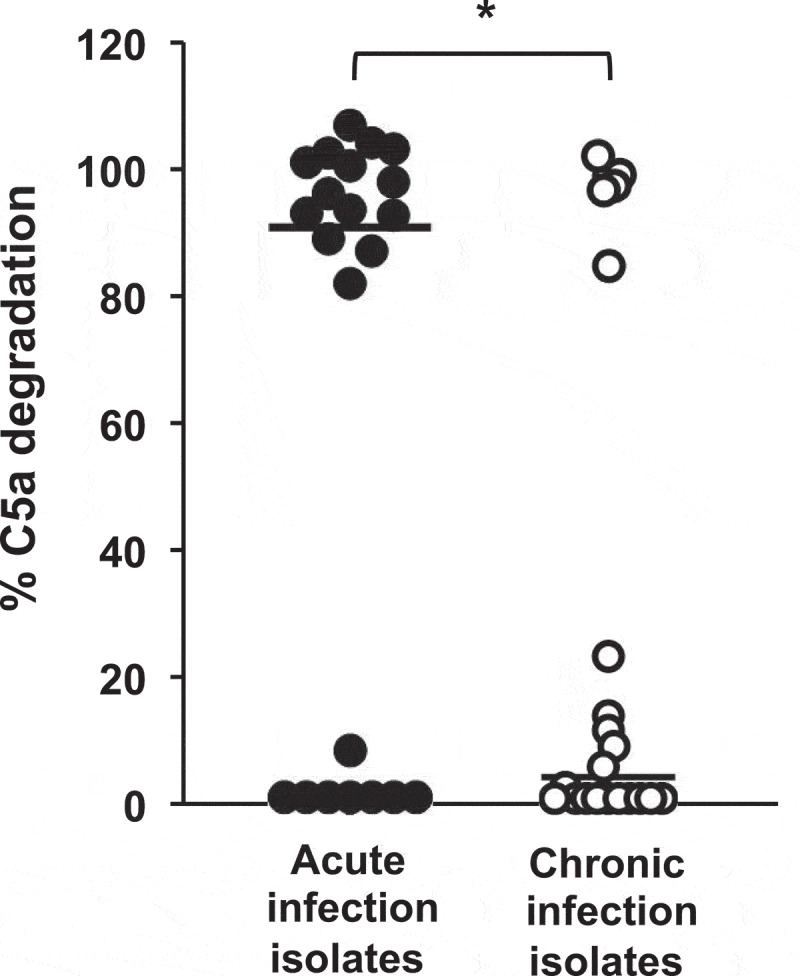
**Analysis of C5a cleavage by *P. aeruginosa* strains isolated from acute and chronic airway infections**. Distribution of the C5a-cleaving phenotype among *P. aeruginosa* strains isolated from acute or chronic airway infections. Recombinant human C5a (20 ng) was incubated with the cell-free supernatants from growth cultures of 44 clinical isolates (22 from acute airway infections and 22 from chronic airway infections). C5a was detected by Western blot using a specific monoclonal antibody. Quantification of the C5a band was carried out by densitometric analysis. Percentage of C5a degradation was calculated with respect to the densitometric value of the untreated C5a. Results are the mean values obtained from three independent experiments for each strain. The median values are plotted with a black bar. *P= 0.02 for the comparison between both groups of isolates (2-tailed t test).

To extend this observation to a higher number of *P. aeruginosa* isolates, we evaluated the distribution of the C5a-cleaving phenotype among 44 genetically unrelated *P. aeruginosa* clinical isolates (22 from acute airway infections and 22 from chronic airway infections). Recombinant human C5a was incubated with the cell-free supernatant of stationary cultures from each isolate and the cleaving activity was determined as described above. Overall, the capacity to degrade C5a was higher among strains from acute infections that among those from chronic infections (P = 0.02; two-tailed t test) (Figure 2). Fourteen isolates (63.6%) from acute infections cleaved C5a almost completely (>80% of C5a degradation with respect to untreated control). Conversely, only five isolates (22.7%) displayed the same ability among those from chronic infections, while most of them (77.3%) exhibited a very low or null C5a-cleaving activity (< 25%).

### Exoproteases AprA and LasB are sufficient to cleave human complement component C5a

To identify the bacterial proteases present in the supernatant of *P. aeruginosa* growth culture involved in the cleavage of C5a, we focused on the two major proteases secreted by this microorganism: alkaline protease (AprA) and elastase B (LasB).

**Figure 3.**
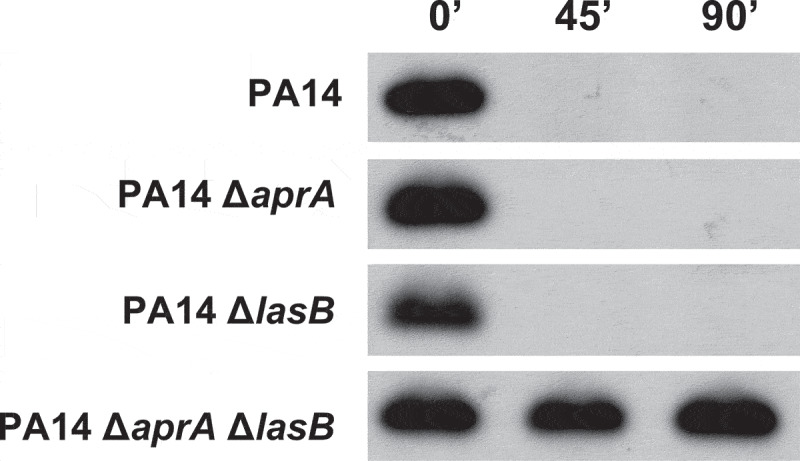
**Contribution of the *P. aeruginosa* exoproteases AprA and LasB to the cleavage of C5a**. Immunoblot analysis of C5a cleavage by *P. aeruginosa*. Purified recombinant human C5a (20 ng) was incubated for different times at 37°C with the cell-free supernatants from stationary growth cultures obtained from *P. aeruginosa* PA14 and the isogenic deficient mutants in AprA (PA14Δ*aprA)*, LasB (PA14Δ*LasB)* or both (PA14Δ*aprA*Δ*LasB*). Proteins were separated and subjected to a Western blot with a mouse monoclonal antibody that recognizes C5a.

To investigate their contribution to the cleavage of C5a, the cell-free supernatant from cultures of the wild-type strain PA14 and the isogenic strains that differed only in the production of AprA (PA14Δ*aprA*), LasB (PA14Δ*lasB*) or both exoproteases (PA14Δ*aprA*Δ*lasB)* were incubated with recombinant human C5a (Figure 3). Immunoblot analysis revealed that the supernatants from PA14 and the single mutants PA14Δ*aprA* and PA14Δ*lasB* were able to cleave C5a similarly. Conversely, the supernatant from the double mutant lacking both proteases was completely unable to cleave C5a, suggesting that the C5a proteolytic activity of *P. aeruginosa* relies predominantly on AprA and LasB.

**Figure 4.**
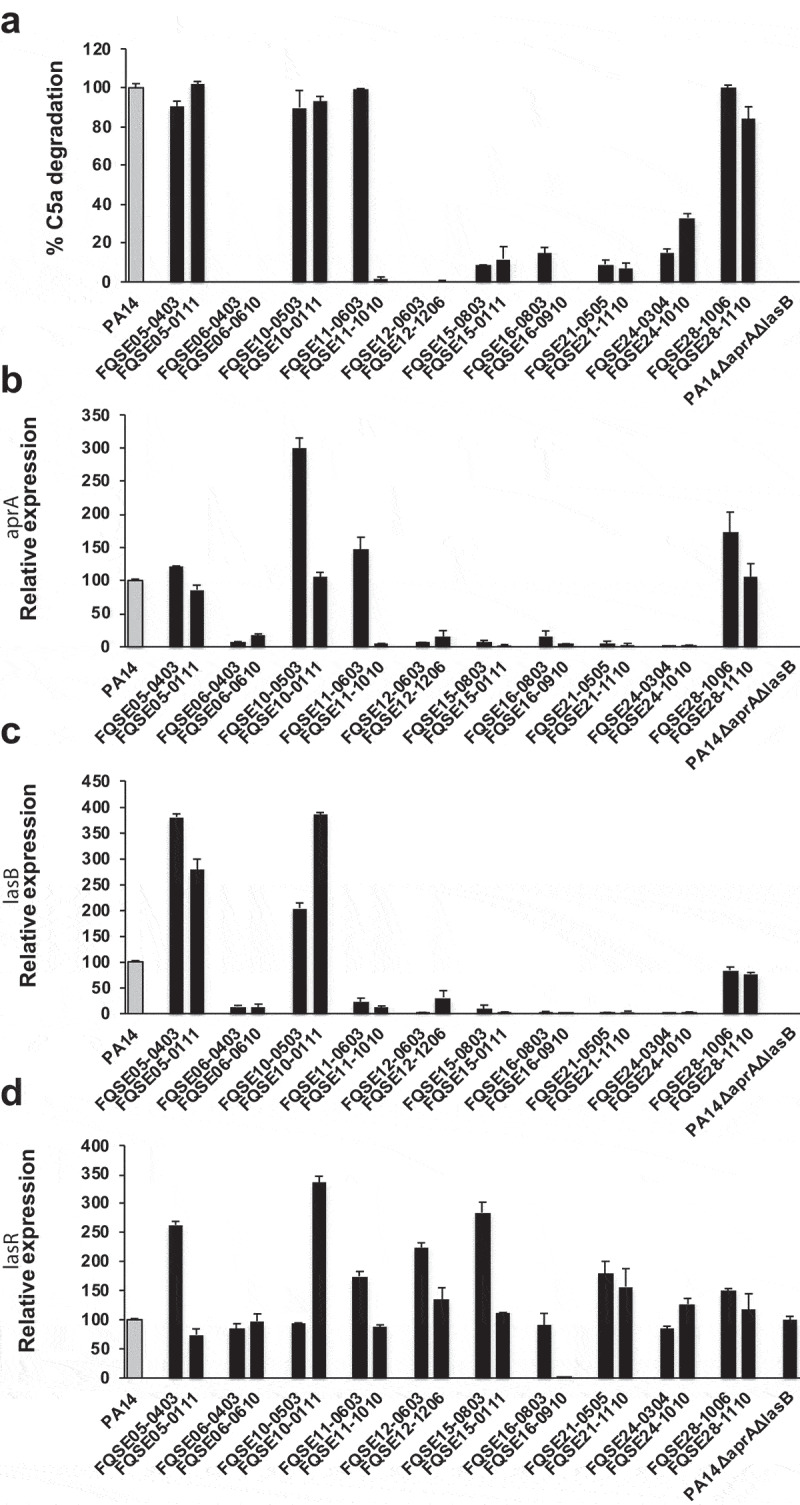
**C5a-cleaving activity and *aprA, lasB* and *lasR* expression of *P. aeruginosa* isolates from CF patients**. (a) Purified recombinant human C5a (20 ng) was incubated with the cell-free supernatant of growth cultures obtained from the CF isolates. The amount of intact C5a was determined by Western blot using a monoclonal antibody that recognizes C5a. Quantification of the C5a band was carried out by densitometric analysis. Percentage of C5a degradation was calculated with respect to the densitometric value of the untreated C5a. Results are the mean values obtained from three independent experiments for each strain. Error bars represent SEMs. (b-d) Relative *aprA* (b) *lasB* (c) and *lasR* (d) expression in *P. aeruginosa* CF isolates grown in LB broth. Gene expression was determined by RT-PCR and normalized using the housekeeping gene *rpsL*. Data are displayed as the percentage of the expression of each gene with respect to PA14 (gray bar). Results are the mean values obtained from three independent experiments. Error bars represent SEMs.

To investigate whether the incapacity to cleave C5a exhibited by the isolates from chronic respiratory infections was associated to a low expression of *aprA* and *lasB*, the C5a-cleaving activity and the expression of *aprA* and *lasB* of a collection of 10 pairs of isolates, including the earliest (early) and the latest (late) isolate recovered from 10 chronically infected CF patients (see Table S1 in the supplementary material), was determined. We found that the expression of *aprA* and *lasB* was lower in the non-C5a cleaving strains compared to strains that cleaved C5a (Figure 4a-c). In fact, reduced expression of *aprA* or *lasB* was correlated with a lower capacity to cleave C5a (P = 0.013 and 0.012 for *aprA* and *lasB*, respectively) (see Fig. S2 in the supplemental material).

### Reduced C5a-cleaving activity is due to mutations in LasR

To gain insight in the underlying mechanism that reduced the expression of *aprA* and *lasB*, the mRNA levels of LasR, a master regulator that positively regulates *aprA* and *lasB*, was determined by real-time PCR. Overall, the level of *lasR* transcripts was similar or higher than in the reference strain PA14 for all strains including those with reduced capacity to cleave C5a and expressing low levels of *aprA* and *lasB* except strain FQSE16-0910, which expressed very low levels of *lasR* (Figure 4d). This observation suggested the existence of nonsynonymous *lasR* mutations that inactivate LasR function. To explore this hypothesis, the sequence of *lasR* from the 20 isolates was determined. We identified sequence variations in all strains that exhibited a reduced capacity to cleave C5a, while the strains that degraded C5a coded for a polypeptide sequence identical to the reference strain PA14. The positions and variants that resulted in a non-C5a cleaving phenotype are shown in Table 1. Six strains, belonging to the same PFGE-type (see supplemental Table S1), harbored a *lasR* variant with a C-to-G change at nucleotide 350 coding for a proline-to-arginine change. Three strains contained substitutions that resulted in stop codons. Two strains contained a frameshift mutation due to a 2 bp deletion at position 206. Finally, strain FQSE11-1010 harbored a G-to-A change at nucleotide 647 coding for an arginine-to-glutamine change in the DNA-binding domain of LasR. This latter strain is of special interest because it is clonally related with the C5a-cleaving strain FQSE11-0603 (early isolate) that was isolated from the same patient 7 years earlier.

**Table 1.** Location of *lasR* sequence variations in non-C5a-cleaving isolates from chronically infected CF patients

Isolate	Nucleotide change	Amino acid change
FQSE 06–0403	C350G	P117R
FQSE 06–0610	C350G	P117R
FQSE 11–1010	G647A	R216Q
FQSE 12–0603	G561A	Q186*
FQSE 12–1206	G561A	Q186*
FQSE 15–0803	C350G	P117R
FQSE 15–1010	C350G	P117R
FQSE 16–0803	G561A	Q186*
FQSE 21–0505	2 bp deletion @ 206 (-AC)	Frameshift
FQSE 21–1110	2 bp deletion @ 206 (-AC)	Frameshift
FQSE 24–0304	C350G	P117R
FQSE 24–1010	C350G	P117R

* Stop codon

**Figure 5.**
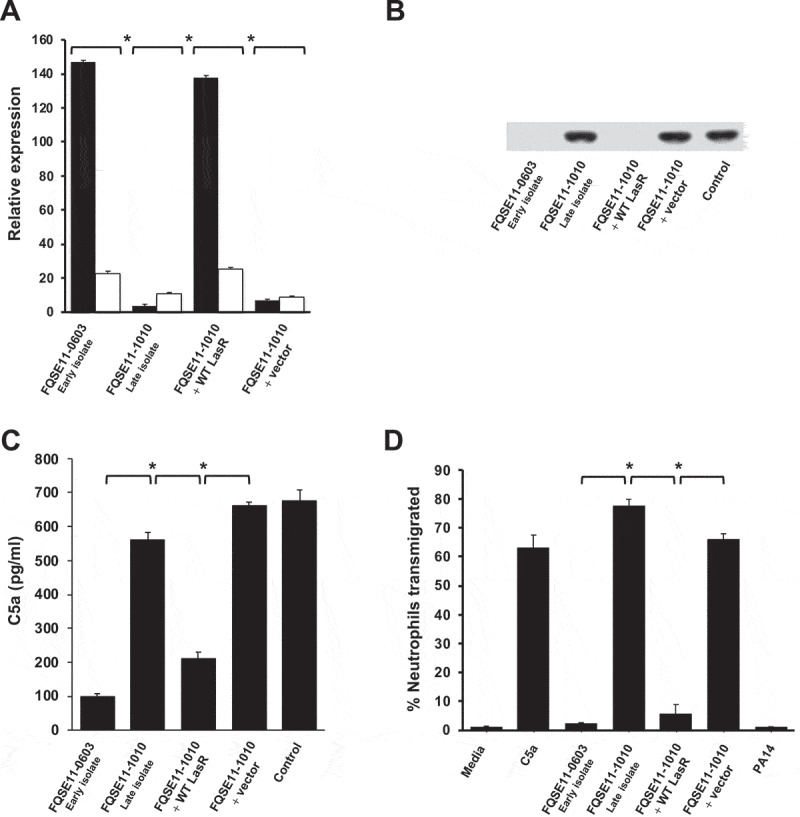
**LasR mutation abolishes AprA/LasB C5a-cleaving activity and enhances neutrophil recruitment**. (a) Relative expression of *aprA* (black columns) and *lasB* (white columns) determined by RT-PCR and normalized using the housekeeping gene *rpsL*. Data are displayed as the percentage of the expression of each gene with respect to PA14. Asterisks indicate significant differences in the expression of both genes between strains *P < 0.05 (2-tailed *t* test). (b) Western blot analysis of C5a cleavage. Purified recombinant human C5a (20 ng) was incubated with LB (control) or the cell-free supernatants from the bacterial stationary cultures in LB. Proteins were separated and subjected to a Western blot with a mouse monoclonal antibody that recognizes C5a. (c) Human BALF was incubated with the cell-free supernatants from the bacterial growth cultures in artificial sputum medium and the amount of C5a was quantified by sandwich ELISA. (d) Human neutrophil transmigration assay with media, purified C5a (100 nM) or C5a treated with cell-free supernatants from the bacterial stationary cultures in LB. Data are the percentage of the neutrophils recovered from the basolateral chamber with respect to total number of neutrophils added to the upper chamber. Results are the mean values obtained from three independent experiments. Error bars represent SEMs. *P < 0.05 (2-tailed *t* test).

To confirm that the mutation of LasR led to the reduced C5a-cleaving capacity of FQSE11-1010 (late isolate), *lasR* from the early isolate FQSE11-0603 was cloned in the late isolate FQSE11-1010. Cloning of *lasR* restored the expression of *aprA* and *lasB* (Figure 5a). Mass spectrometry analysis of the cell-free supernantants of growth cultures containing equal numbers of cells of each strain confirmed the absence of AprA and LasB in the late isolate and their presence in the early and the complemented strain (data not shown).

Furthermore, complementation of the late isolate with *lasR* from the early isolate also restored the C5a-cleaving activity of the late isolate FQSE11-1010 to the same levels observed in the early isolate FQSE11-0603 (Figure 5b).

This result was further confirmed using human BALF instead of purified C5a and growing the strains in artificial sputum medium to the same cell density. The cell-free supernatant of the culture from the early isolate and from the late isolate complemented with *lasR* from the early isolate were able to degrade BALF C5a, while the supernatant from the late isolate was not (Figure 5c).

If the mutation of *lasR* impairs the AprA/LasB-mediated cleavage of C5a, we hypothesized that LasR deficient variants should increase neutrophil recruitment. To evaluate this idea, we performed a neutrophil transmigration assay to assess the impact of LasR deficiency on neutrophil recruitment and chemotaxis in vitro. We used recombinant human C5a treated with the cell-free supernatants of cultures from the set of strains from patient FQSE11 and determined the effects on neutrophil chemotaxis and transmigration. C5a treated with the supernatant of the late isolate FQSE11-1010 induced 33- and 14-fold greater neutrophil transmigration, respectively, compared to C5a treated with the supernatant from the early isolate or the late isolate complemented with functional *lasR* (all P < 0.01) (Figure 5d). Thus, *P. aeruginosa* variants lacking AprA and LasB function due to mutation of the transcriptional regulator LasR may induce intense neutrophil recruitment because of their incapacity to cleave C5a.

To investigate when LasR mutant FQSE11-1010 emerged in the lung of patient FQSE11, the C5a cleaving capacity and the sequence of *lasR* of six additional clonally related longitudinal isolates from the same patient were determined (see Fig. S3 in the supplemental material). All isolates collected between June 2003 and May 2010 cleaved C5a and presented a *lasR* sequence identical to the reference strain PA14, indicating that, in this particular patient, *lasR* mutation occurred at least after more than seven years in the lung of FQSE11 patient.

## Discussion

The results described in this report provide evidence that *P. aeruginosa* AprA and LasB are able to cleave directly C5a. This capacity is lost by the microorganism during the adaption to the CF lung environment due to mutations in *lasR* that result in a nonfunctional regulator.

It was previously established that purified exoproteases AprA and LasB cleave early complement components C1 and C2 and the central component C3 [21,22], preventing the activation of C5 into C5a and C5b. Our results using culture filtrates from PA14 and the isogenic mutants deficient in AprA, LasB or both demonstrate that both exoproteases, not only have the potential to reduce the formation of C5a but also to cleave directly this important anaphylatoxin, which is essential for protection against *P. aeruginosa* lung infection [23,24]. It would be interesting to confirm the C5a-cleaving activity of the purified AprA and LasB in vitro.

This previously unrecognized C5a-cleaving activity of both proteases may be crucial for *P. aeruginosa* strains causing acute infections, which are usually resistant to the bactericidal effect of the complement and are mainly cleared by opsonophagocytosis by the leukocytes recruited to the site of the infection [25]. In fact, our results demonstrate that most of the strains isolated from acute airway infections cleave C5a, which could represent a strategy, previously described in other pathogens such as group A and group B *Streptococcus* [26,27] that *P. aeruginosa* exploits to avoid the chemotactic effects of this molecule. On the other hand, our findings indicate that the C5a generated by the strains from chronic respiratory infections, in most cases, is not cleaved and probably accumulates in the respiratory tract. This idea is supported by the high levels of C5a found in the CF lung fluids colonized by *P. aeruginosa* [9–11].

We made use of a well-characterized set of strains isolated from 10 chronically infected CF patients to investigate the genetic changes that led to the non-C5a-cleaving phenotype. In this collection, the incapacity to cleave C5a was associated to a low expression of *aprA* and *lasB* caused by mutations in *lasR. P. aeruginosa* isolates from CF patients frequently contain mutations in *lasR* [28–31], although some of them render a protein with residual activity [32]. Consistent with these studies, 60% of our CF isolates harbored a mutation that resulted in a regulator with a very low or null activity. We identified a novel LasR variant R216Q located in the DNA-binding domain of LasR. This LasR variant had a dramatic effect on the expression of *aprA* and *lasB* giving rise to the C5a-cleaving deficient isolate FQSE11-1010. The substitution of the proline at position 117, located in the signal-binding domain of the protein, by an arginine was the most common variant in our isolates. The substitution of the same proline by a leucine resulted in a functional LasR variant with 50% of its relative activity in *P. aeruginosa* PAO1 [32].

The emergence of LasR variants in the chronically infected CF lung have been associated with a worsening of the disease [31], but a causal relationship is still under investigation. It has been shown that LasR-deficient isolates from CF patients induce exaggerated host inflammatory responses and promote neutrophil recruitment due to the loss of the LasB cytokine-proteolytic activity [33]. More recently, Hennemann et al demonstrated that LasR-deficient strains enhanced neutrophil adhesion due to the loss of ICAM-1 degradation by LasR-regulated proteases [34]. Our findings complement these previous studies on the potential detrimental effects of the LasR mutations. Thus, it is likely that the loss of the AprA/LasB-dependent proteolysis of C5a exhibited by the *lasR*-mutant isolates may contribute to the intense neutrophilia observed in the lung fluids of the CF patients [9,28] and in the previous studies with mice challenged with LasR-deficient mutants [33,34]. It is noteworthy that, in addition to the neutrophil chemotactic activity, C5a also induces the expression of the neutrophil integrin that mediates the attachment to ICAM-I, facilitating their migration to the lung. Altogether, the lack of C5a, ICAM-I and cytokine proteolytic activity exhibited by the lasR deficient variants might explain why the emergence of LasR deficient variants in the CF lung microbiome is associated to a poor prognostic and to the increased episodes of acute exacerbations which are typically characterized by an intense lung neutrophilia [31].

In addition, our results extend the knowledge on the possible negative consequences of LasR deficiency. Indeed, given the well-established detrimental impact of the excessive C5a on the function of the neutrophils [7,8], the key inflammatory cells in CF, we speculate that the non-cleaving C5a phenotype acquired by the LasR mutants may account for the general dysfunction of neutrophils that predispose toward increased inflammation and reduced bacterial clearance described in CF patients [35].

In summary, we have identified complement C5a as a novel substrate for the exoproteases AprA and LasB. We have also demonstrated that most isolates from chronically infected CF patients lose the capacity to inactivate C5a because of mutations in LasR, a specific feature of each strain that may represent a turning point in the course of the CF infection. This new finding illustrates that *P. aeruginosa* has evolved diverse mechanisms to modulate the host inflammatory response.

## Supplementary Material

Supplemental Material

## Data Availability

The authors confirm that the data supporting the findings of this study are available within the article [and/or] its supplementary materials.
