# Supplementary material for: Pseudomonas aeruginosa adaptation in cystic fibrosis patients increases C5a levels and promotes neutrophil recruitment
Source: Virulence. 2022 Jan 30;13(1):215–24. doi: 10.1080/21505594.2022.2028484 (PMC8802900; doi:10.1080/21505594.2022.2028484)
Supplement: Supplemental Material [file KVIR_A_2028484_SM2429.zip › supplementary/Supplementary material.docx]

**FIGURE S1. Analysis of the production of C5a by *P. aeruginosa* reference strain PA14.**

(A). Bacterial cells from PA14 were incubated in human serum (10 %) and the amount of C5a produced was determined at different times by Western blot using a monoclonal antibody that recognizes C5a. Quantification of the C5a band was carried out by densitometric analysis.

Results are the mean values and the standard deviation obtained from three independent experiments.

**Figure S2. Association of *aprA* and *lasB* expression with C5a-cleaving activity.**

Pearson correlation analysis of the expression of *aprA* (A) and *lasB* (B) with C5a-cleaving activity of 20 strains isolated from chronically infected CF patients. Each dot represents an individual strain. C5a-cleaving activity is expressed as the percentage of C5a degradation calculated as described in Fig. 4A. Gene expression was calculated as described in Figures 4B and C.

P values (P = 0.013 and 0.012 for *aprA* and *lasB*, respectively) were determined with the two-tailed t test. Spearman correlation R was 0.5442 and 0.5479 with 95% confidence intervals (0.1201 to 0.8003) and (0.1254 to 0.8022), respectively.


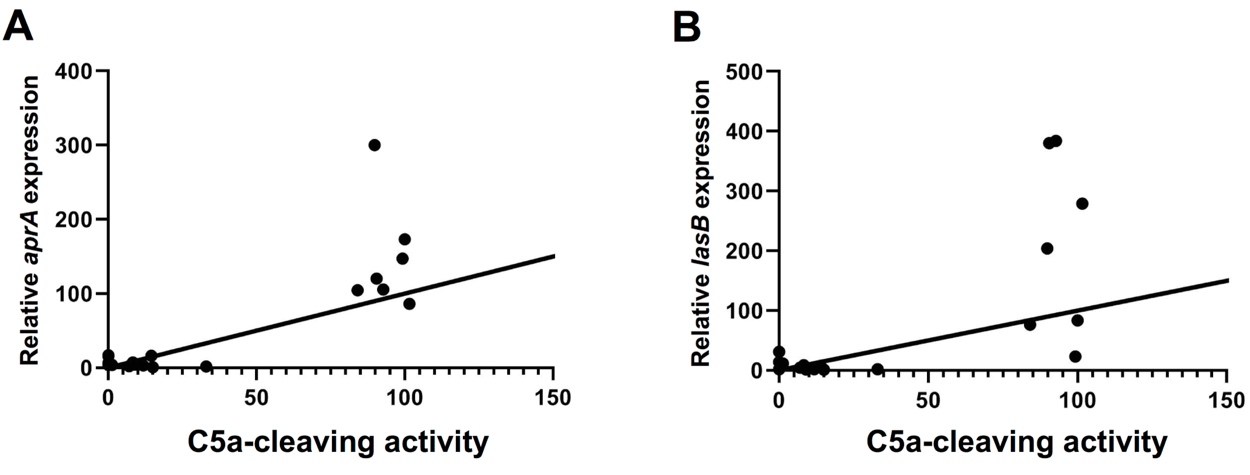


**Figure S3. C5a-cleaving activity and LasR sequence of clonally related longitudinal *P. aeruginosa* isolates from CF patient FQSE11*.***

Purified recombinant human C5a (20 ng) was incubated with LB (control) or the cell-free supernatants of stationary cultures from clonally-related *P. aeruginosa* isolates collected over seven years from the same patient. Proteins were separated and subjected to a Western blot with a mouse monoclonal antibody that recognizes C5a. LasR amino acid sequence variants with respect to the sequence of the reference strain PA14 (Wild type) are indicated below each isolate.


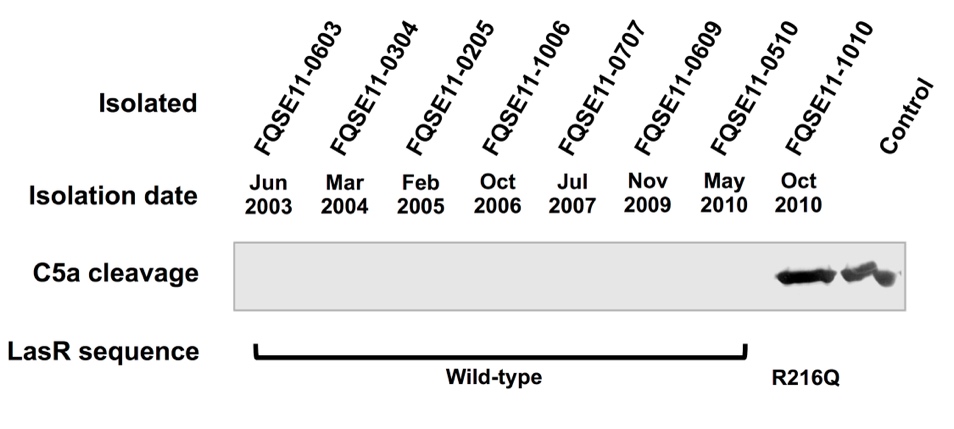


**Table S1. *P. aeruginosa* sequential isolates from patients with cystic fibrosis used in this study.**

| Patient | Isolate | PFGE Clone^a^ | MLST^b^ | Isolation date |
| --- | --- | --- | --- | --- |
| FQSE 05 | 0403 | FQSE-E | 1108 | April 2003 |
|  | 0111 | FQSE-E | 1108 | January 2011 |
| FQSE 06 | 0403 | FQSE-A | 274 | April 2003 |
|  | 0610 | FQSE-A | 274 | June 2010 |
| FQSE 10 | 0503 | FQSE-A | 274 | May 2003 |
|  | 0111 | FQSE-A | 274 | January 2011 |
| FQSE11 | 0603 | FQSE-K | 701 | June 2003 |
|  | 0304 | FQSE-K | 701 | March 2004 |
|  | 0205 | FQSE-K | 701 | February 2005 |
|  | 1006 | FQSE-K | 701 | October 2006 |
|  | 0707 | FQSE-K | 701 | July 2007 |
|  | 0609 | FQSE-K | 701 | June 2009 |
|  | 0510 | FQSE-K | 701 | May 2010 |
|  | 1010 | FQSE-K | 701 | October 2010 |
| FQSE 12 | 0603 | FQSE-C | 299 | June 2003 |
|  | 1206 | FQSE-C | 299 | December 2006 |
| FQSE 15 | 0803 | FQSE-A | 274 | August 2003 |
|  | 0110 | FQSE-A | 1089^C^ | January 2010 |
| FQSE 16 | 0803 | FQSE-M | 1073 | August 2003 |
|  | 0910 | FQSE-M | 1073 | September 2010 |
| FQSE 21 | 0505 | FQSE-I | 1109 | May 2005 |
|  | 1110 | FQSE-I | 1109 | November 2010 |
| FQSE 24 | 0304 | FQSE-A | 1089 | March 2004 |
|  | 1010 | FQSE-A | 1089 | October 2010 |
| FQSE 28 | 1006 | FQSE-J | 1071 | October 2006 |
|  | 1110 | FQSE-J | 1071 | November 2010 |

a Clonal relatedness evaluated by pulsed-field gel electrophoresis (PFGE).

b Clonal relatedness evaluated by multilocus sequencing typing (MLST).

c ST1089 derives from ST274.

**Table S2.** Primers used in this study.

| Primer | Sequence (5’-3’) |
| --- | --- |
| RT-AprA-F | TCGGTGATGAGCTACTGGGA |
| RT-AprA-R | AGAAGTCCAGGGTGTCGTTG |
| RT-LasB-F | TGTCCAAACTCCCCAGCAAG |
| RT-LasB-R | GCGATGTTGGCGACGAAATG |
| RT-LasR-F | TCCATCTACCAGACGCGAAAG |
| RT-LasR-R | GTTTGCTGACCGGATGTTCG |
| RT-Rpsl-F | GCTGCAAAACTGCCCGCAACG |
| RT-Rpsl-R | ACCCGAGGTGTCCAGCGAACC |
| LasR Seq-F | TAGAGTGGGCTGACTGGACA |
| LasR Seq-R | CAGTCGTTTCGAGAATGGCG |
| LasR-F | CCCCGAATTCTAGCGCTATGGCCTTGGTTG |
| LasR-R | CCCAAGCTTGCAAGATCAGAGAGTAATAAGACCC |
